# The Impact of Autism Spectrum Disorder and Alexithymia on Judgments of Moral Acceptability

**DOI:** 10.1037/abn0000076

**Published:** 2015-08

**Authors:** Rebecca Brewer, Abigail A. Marsh, Caroline Catmur, Elise M. Cardinale, Sarah Stoycos, Richard Cook, Geoffrey Bird

**Affiliations:** 1 MRC Social, Genetic and Developmental Psychiatry Centre, Institute of Psychiatry, King’s College London; 2 Department of Psychology, Georgetown University; 3 Department of Psychology, University of Surrey; 4 Department of Psychology, Georgetown University; 5 Department of Psychology, University of Southern California; 6 Department of Psychology, City University London; 7 MRC Social, Genetic and Developmental Psychiatry Centre, Institute of Psychiatry, King’s College London, and Institute of Cognitive Neuroscience, University College London

**Keywords:** alexithymia, morality, autism, emotion identification, empathy

## Abstract

One’s own emotional response toward a hypothetical action can influence judgments of its moral acceptability. Some individuals with autism spectrum disorder (ASD) exhibit atypical emotional processing, and moral judgments. Research suggests, however, that emotional deficits in ASD are due to co-occurring alexithymia, meaning atypical moral judgments in ASD may be due to alexithymia also. Individuals with and without ASD (matched for alexithymia) judged the moral acceptability of emotion-evoking statements and identified the emotion evoked. Moral acceptability judgments were predicted by alexithymia. Crucially, however, this relationship held only for individuals without ASD. While ASD diagnostic status did not directly predict either judgment, those with ASD did not base their moral acceptability judgments on emotional information. Findings are consistent with evidence demonstrating that decision-making is less subject to emotional biases in those with ASD.

## Abstract (significance statement)

This study suggests that typical people base their judgments of the moral acceptability of behavior on their emotional response to that behavior. People with autism spectrum disorder do not seem to use emotional information and may rely more on rules to judge moral acceptability.

Moral reasoning plays a critical role in human societies, resting upon moral principles that prescribe how individuals ought to behave. Individual differences in moral ideology may lead to the adoption of different moral principles, however, with subsequent impact upon moral reasoning. For example, individuals may be more concerned with the moral acceptability of behavior that is undertaken (deontologists), or with the consequences of that behavior (utilitarians/consequentialists). Philosophers have debated the role of emotions in moral reasoning; although some argue that morality is a purely rational process, based upon deliberative reasoning (Cudworth, 1996; Kant, 1785/1965), others emphasize the role of emotions (Hume, 1777/1960; Prinz, 2004). It is now generally accepted that both emotional and rational processes contribute to moral decision-making (Cushman, Young, & Greene, 2010; Ugazio, Lamm, & Singer, 2012). The dual-process model of morality (Greene, Sommerville, Nystrom, Darley, & Cohen, 2001; Greene, Nystrom, Engell, Darley, & Cohen, 2004) posits that individuals attend to their own emotional response toward engaging in different behaviors, as well as deliberating upon the outcomes of these behaviors, to judge their moral acceptability.

Consistent with emotions being involved in moral decision-making, automatic emotional reactions to victims’ emotional states influence moral judgments (Haidt, 2001), and lead to condemnation of moral violations (Decety & Cacioppo, 2012; Pizarro, 2000). Aversive emotional reactions to such behaviors lead to a judgment that any deliberate action causing distress is immoral (Avramova & Inbar, 2013; Haidt, 2001). Thus, emotional responses to immoral behavior may arise through two routes; direct emotional response to the behavior itself, and empathic reaction to the distress the behavior elicits in its victim (Miller & Cushman, 2013; Pizarro, 2000). Neurological evidence also suggests that moral reasoning recruits brain regions involved in empathy and emotion recognition in oneself and others (Bzdok et al., 2012; Greene, 2003; Moll et al., 2002). Conditions associated with impaired recognition of one’s own emotions may also be associated with atypical moral acceptability judgments, therefore. Crucially, the degree to which impaired recognition of one’s own emotion affects moral reasoning should depend on the degree to which one relies on emotional versus deliberative reasoning when making moral judgments.

Alexithymia is a subclinical trait associated with difficulties identifying and describing one’s own emotions (Nemiah, Freyberger, & Sifneos, 1976). Consistent with a role for the identification of one’s own emotion during moral reasoning, increased alexithymia is associated with more utilitarian decision-making (Patil & Silani, 2014b), and increased perceived permissibility of accidentally harming others (Patil & Silani, 2014a). Decreased ability to recognize emotions in oneself therefore affects moral decisions, in line with the dual-process theory of morality (Greene et al., 2001; Greene et al., 2004).

It is widely reported that individuals with autism spectrum disorder (ASD) exhibit difficulties recognizing their own emotions (Hill, Berthoz, & Frith, 2004; Rieffe, Meerum Terwogt, & Kotronopoulou, 2007), and empathizing with others (e.g., Baron-Cohen & Wheelwright, 2004), suggesting moral reasoning impairments should also be a feature of ASD. Evidence for atypical moral judgments in ASD populations is mixed, however (Gleichgerrcht et al., 2013; Moran et al., 2011; Li, Zhu, & Gummerum, 2014; Schneider et al., 2013; Zalla, Barlassina, Buon, & Leboyer, 2011). Co-occurring alexithymia in ASD may explain this inconsistency.

Alexithymia characterizes under 10% of the typical population, but approximately 50% of the ASD population (Berthoz & Hill, 2005; Hill et al., 2004). Alexithymia and ASD are distinct, however; alexithymia is neither necessary nor sufficient for an ASD diagnosis, nor is it universal among individuals with ASD. Similarly, co-occurring alexithymia is not specific to ASD; numerous clinical populations (e.g., eating disorders, panic disorder and substance abuse; Grynberg et al., 2012) also co-occur with alexithymia. Recent research demonstrates that, where observed, empathy deficits and emotion recognition impairments in ASD are explained by co-occurring alexithymia, not ASD per se (Bird et al., 2010; Cook, Brewer, Shah, & Bird, 2013; Heaton et al., 2012; Bird & Cook, 2013). Given these findings, and the contribution of emotional identification and empathy to moral reasoning, it is possible that the atypical moral reasoning observed in some individuals with ASD is a product of alexithymia and unrelated to ASD itself. The current study tested this hypothesis, investigating the separate contribution of alexithymia and ASD symptom severity to moral judgments.

## Method

### Participants

Twenty-five individuals (six female) with, and 22 individuals (five female) without a diagnosis of ASD participated in this study. Twenty-four typical individuals initially participated, but two were removed to match the groups according to alexithymia, measured by the Toronto Alexithymia Questionnaire (TAS-20; Bagby, Parker, & Taylor, 1994), *t*(45) = 1.56, *p* = .128, 95% confidence interval (CI) [14.32, −1.88] (control *M* = 50.14, *SD* = 16.03; ASD *M* = 56.36, *SD* = 10.30). The TAS-20 has high reliability and validity (Parker, Taylor, & Bagby, 2003) and includes items such as “I am often confused about what emotion I am feeling” and “It is difficult for me to find the right words for my feelings,” answered on a scale from 1 (*does not describe me well*) to 5 (*describes me very well*). The ASD and control groups were also matched according to age, *t*(45) = .885, *p* = .381, *d* = .53, 95% CI [10.64, −4.14] (control *M* = 31.27, *SD* = 12.16; ASD *M* = 34.52, *SD* = 12.88), gender, χ^2^(1) = .01, *p* = .918, and IQ, measured using the Wechsler Abbreviated Scale of Intelligence (Wechsler, 1999), *t*(45) = .061, *p* = .951, 95% CI [10.04, −9.45] (control *M* = 106.86, *SD* = 16.20; ASD *M* = 107.16, *SD* = 16.85).

The Autism-Spectrum Quotient (AQ; Baron-Cohen, Wheelwright, Skinner, Martin, & Clubley, 2001) assessed ASD symptom severity in all participants. AQ scores were significantly higher in the ASD (*M* = 26.63, *SD* = 11.68) than control group (*M* = 18.85, *SD* = 8.60), *t*(45) = 3.36, *p* = .002, 95% CI [14.83, 3.72]. Current functioning of all individuals in the ASD group was assessed with the Autism Diagnostic Observation Schedule (ADOS) Module 4 (Lord et al., 2000). ADOS scores meeting criteria for ASD may be categorized as indicative of either autism or autism spectrum. Of the 25 participants with a clinical diagnosis of ASD (assessed by independent clinicians, according to *DSM–IV* criteria), 21 also met the ADOS criteria for ASD (13 for autism, eight for autism spectrum). Although four of the individuals in the ASD group did not meet criteria for ASD according to the ADOS, they received diagnoses from independent clinicians and scored above cut-off for autism on the AQ. These individuals were included in the reported analyses but were not outliers on any analysis, and their exclusion did not alter the pattern of results.

### Procedure

A previously validated task assessed moral judgments (Marsh & Cardinale, 2012). Participants viewed 100 emotive statements, equally divided into those evoking happiness, sadness, fear, disgust, and anger. Statements include “I bought you a present” (happiness), “I do not want to be friends any more” (sadness), “I could easily hurt you” (fear), “I never wash my hands” (disgust), and “I broke your phone on purpose” (anger). Each statement was presented once, with order randomized across participants. Participants were required to rate the moral acceptability of saying each statement to another person, ranging from 1 (*never acceptable*) to 4 (*always acceptable*). Ability to identify the evoked emotion was assessed by presenting the same statements in a random order, and requiring participants to identify their own emotional response to each statement, from happiness, sadness, disgust, anger, and fear.

Five moral acceptability scores were calculated for each participant by taking the mean rating for each of the emotion-inducing categories. A Global Morality score, where higher scores indicate more severe difficulties in judging moral acceptability, was calculated by taking the mean moral acceptability scores for the five statement types, with happiness acceptability ratings reverse-scored.

When assessing identification of emotion, scores were assessed with respect to the typical validation sample reported in Marsh and Cardinale (2012). An “error” score indexed the frequency with which participants selected an atypical emotion, whereby increasing values indicate less typical performance, using the following equation.

$$Error\;Score\;=\;{\sum (\text{Number of Correct Responses}\;\text{ −}\;\text{ Perfect Performance)}}^{2}$$

Perfect performance was 20 responses of the statement emotion, and zero responses of all other emotions.

### Data Analysis

*T* Tests determined whether the ASD and alexithymia-matched control groups differed in Global Morality score. Correlation analyses determined the relationship between ASD symptom severity, alexithymia, and moral acceptability judgments, in the full sample, and the ASD and control groups separately. Fisher’s *r*-to-*z* transformations compared the correlation coefficients in the two groups. Hierarchical regression analyses (conducted separately in the control and ASD groups) determined whether alexithymia or ASD symptom severity predicted moral acceptability judgments once age, gender, depression, and anxiety were controlled for, and whether each could predict the dependent variables after the other was controlled for. It is necessary to perform hierarchical regressions with alexithymia and ASD symptom severity entered in both possible orders to independently investigate the effect of each, after controlling for the other, because of colinearity. Analyses that do, and do not, control for alexithymia when assessing the impact of ASD (and vice versa) allow for the potential overlap in measures of each construct to be accounted for. If the AQ (a putative measure of ASD symptom severity) also taps into some features of alexithymia, controlling for alexithymia when assessing the impact of ASD using the AQ will provide a more “pure” measure of ASD traits, unconfounded by alexithymia. If alexithymia is a feature of ASD (which we suggest is incorrect), however, then the above analyses using raw AQ scores without controlling for alexithymia would be judged to be more appropriate. A regression analysis determined whether ASD group moderated the relationship between alexithymia and moral judgments. Finally, the extent to which emotional identification predicted moral acceptability judgments was investigated in each group using correlation analyses.

## Results

The ASD (*M* = 1.79, *SD* = .45) and alexithymia-matched control groups (*M* = 1.92, *SD* = .58) did not differ in Global Morality score, *t*(45) = .850, *p* = .400, 95% CI [.17, −.43], or individual morality scores (see Table 1). Global Morality score was uncorrelated with ASD symptom severity, measured by AQ, *r* = .220, *p* = .137, but was significantly related to alexithymia, *r* = .391, *p* = .007. No morality score for the individual emotional categories correlated significantly with ASD symptom severity, while alexithymia significantly predicted morality judgments of statements eliciting happiness, *r* = −.377, *p* = .009, fear, *r* = .390, *p* = .007 and anger, *r* = .390, *p* = .007. Correlations between moral acceptability judgments for the different statement types are shown in Table 2, for the full sample and the control and ASD groups separately.[Table-anchor tbl1][Table-anchor tbl2]

**Figure 1 fig1:**
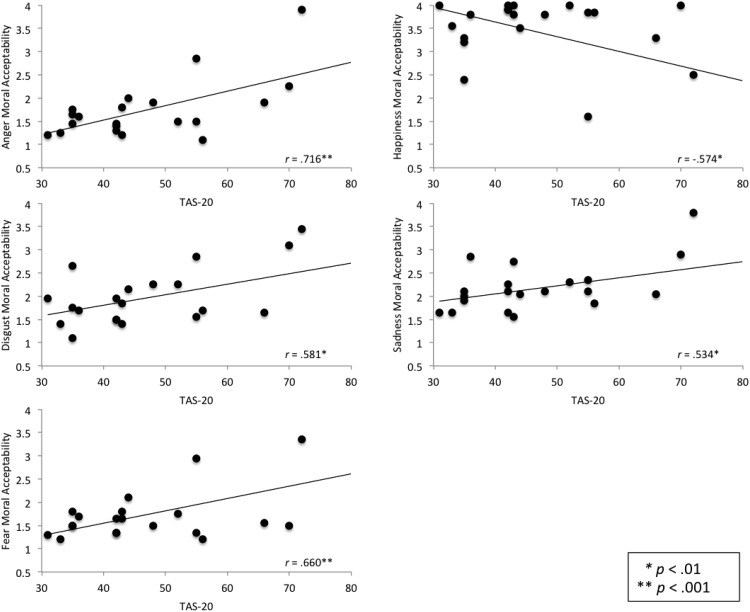
Correlations between alexithymia and moral acceptability judgments for anger, disgust, fear, happiness, and sadness-inducing statements in the control group. TAS-20 = Toronto Alexithymia Questionnaire.

To determine whether the relationship between alexithymia and moral acceptability ratings varies across the ASD and control groups, correlational analyses were conducted in the groups separately. Alexithymia correlated significantly with Global Morality score in the control sample, *r* = .716, *p* < .001, but not the ASD sample, *r* = −.053, *p* = .802. A Fisher’s *r*-to-*z* transformation indicated that the two correlations differed significantly from each other (*Z* = 3.04, *p* = .002). Alexithymia was also significantly associated with moral acceptability judgments for all statement types in the control group (see Figure 1), but not with moral acceptability judgments for any of the emotion categories in the ASD group.[Fig-anchor fig1]

Hierarchical regression analyses were conducted separately in the ASD and control groups. In the control group, alexithymia significantly predicted Global Morality judgments over and above age, gender, depression, and anxiety, regardless of the order alexithymia and ASD symptom severity were entered into the regression model (see Table 3), whereas ASD symptom severity did not significantly predict Global Morality when alexithymia was also included in the model. In the ASD group, neither alexithymia nor ASD symptom severity predicted Global Morality.[Table-anchor tbl3]

Linear regression, with the independent variables alexithymia, ASD group, and their interaction term (Alexithymia × ASD Group), determined whether ASD group moderated the relationship between alexithymia and moral acceptability judgments. Although alexithymia was (β = .311, *t* = 2.237, *p* = .031) and ASD group was not (β = −.203, *t* = −1.565, *p* = .125) a significant predictor of global morality score, the interaction term significantly predicted morality judgments (β = −.361, *t* = −2.673, *p* = .011). ASD group therefore moderated the effect of alexithymia on moral acceptability judgments.

Finally, correlation analyses compared the relationship between emotion identification typicality and moral acceptability judgments in each group. In the control group, emotion identification scores correlated with Global Morality scores, *r* = .741, *p* < .001, whereas these scores were not correlated in the ASD group, *r* = .093, *p* = .657. A Fisher’s *r*-to-*z* transformation indicated that the relationship was significantly stronger in the control than ASD group (*Z* = 2.74, *p* = .003).

## Discussion

It is widely suggested that both deliberative reasoning and emotional responses contribute to judgments concerning the moral acceptability of behavior. If emotions shape moral judgments, impairments identifying one’s own emotional responses, such as in alexithymia, may cause atypical moral acceptability judgments (Patil & Silani, 2014a, 2014b), with the degree to which emotion identification impacts upon moral reasoning, dependant upon the relative influence of deliberative reasoning and emotional processes. We tested the hypothesis that alexithymia, rather than ASD per se, is related to moral judgments through its impact upon emotion identification. The hypothesis was partly supported; although ASD did not affect judgments of moral acceptability, it moderated the relationship between alexithymia and these judgments.

In typical individuals, alexithymia was associated with atypical moral acceptability judgments. Individuals with more severe alexithymia considered it less acceptable to induce happiness in others, and more acceptable to induce sadness, fear, disgust, and anger. In individuals with ASD, however, alexithymia did not predict moral acceptability judgments. This differential pattern of results suggests the reliance on two different strategies when making judgments of moral acceptability. This conclusion was supported by analyses comparing the identification of emotion with moral acceptability judgments; whereas the degree to which emotion identification was (a)typical correlated with moral acceptability judgments in those without ASD, these were uncorrelated in individuals with ASD. Although typical individuals judged the moral acceptability of emotion-evoking statements based on the emotion likely to be evoked, and alexithymia, characterized by reduced emotion identification, negatively impacted on this process, those with ASD did not rely on emotion judgments when judging moral acceptability.

In line with the dual process model of morality, results indicated that individuals with ASD base their moral judgments on factors other than their emotional responses. Decreased reliance on emotion in those with ASD is consistent with previous reports of reduced emotional biases during decision-making in this population (Damiano, Aloi, Treadway, Bodfish, & Dichter, 2012; De Martino, Harrison, Knafo, Bird, & Dolan, 2008). These findings have been explained within the context of a “two-systems” model of human judgment (Evans, 2003), in which both intuitive and analytic processes interact. Crucially, the intuitive process is subject to contextual emotional information (Kahneman, 2003). Previous work has identified a role for the amygdala in such emotionally biased decision-making (De Martino, Kumaran, Seymour, & Dolan, 2006; Kahneman & Frederick, 2007), suggesting that decision-making in ASD is less subject to emotional information due to reduced activation or connectivity of the amygdala (De Martino et al., 2008). Within the context of moral acceptability judgments, individuals with ASD may rely on learnt social norms rather than emotional information, in line with evidence that they rely more on rule-based than emotional rationales when evaluating their own hypothetical prosocial behavior (Jameel, Vyas, Bellesi, Cassell, & Channon, 2015). Alternatively, variance in understanding of causal relationships may predict moral judgments in ASD; reduced understanding of the consequences of one’s actions may cause some behaviors to be perceived as morally acceptable until negative effects are observed.

Regarding the lack of a direct effect of ASD in moral reasoning, the current task makes limited demands on theory of mind (ToM; representing others’ mental states). Moral reasoning may require ToM when a victim is harmed mentally but not physically, or an agent’s intention (e.g., to help) does not match the outcome of their behavior (e.g., harming another; Moran et al., 2011). ToM deficits in ASD (Happé, 1994) may cause atypical moral judgments in such situations, particularly in individuals with more severe ToM impairments.

It should be noted that, although alexithymia is not a necessary diagnostic criterion for ASD, diagnostic instruments often include limited measures of emotional competence. This makes it crucial to control for alexithymia when assessing the impact of ASD, and for ASD when assessing the impact of alexithymia. The current study measured ASD symptom severity in all participants using the AQ. Although AQ correlates highly with other measures of ASD severity (e.g., ADOS; Brugha et al., 2012), it is possible that correlations with ASD symptom severity may vary with measurement instrument. Finally, although we screened for ASD traits in the typical sample using the AQ, future studies should confirm the absence of ASD using the ADOS in the typical group.

In conclusion, these findings add to existing literature on alexithymia and moral reasoning in nonclinical populations (Patil & Silani, 2014a, 2014b), suggesting that difficulties in emotional identification, and possibly empathy, not only alter responses to others’ emotions, but also the emotions one elicits in others; increased alexithymia may increase the tendency to cause distress to others during social interactions. Moral behavior is crucial for developing and maintaining social relationships, meaning atypical moral judgments may add to the social difficulties experienced by individuals with alexithymia. The differential results in typical and ASD individuals suggest the relationship between alexithymia and morality is complex, however. As alexithymia co-occurs with several clinical conditions (Grynberg et al., 2012), it is necessary to investigate this relationship across multiple populations. If alexithymia predicts moral judgments in disorders, screening for alexithymia may contribute to decreasing the proportion of individuals with mental health issues currently in the criminal justice system. Systematic examination of the role of alexithymia across a number of clinical conditions is therefore warranted to fully characterize moral reasoning in individuals with psychiatric conditions.

## Figures and Tables

**Table 1 tbl1:** Means, Standard Deviations, and t Test for Group Difference for Moral Acceptability Judgments for the Individual Statement Types

Statement type	Control, mean (*SD*)	ASD, mean (*SD*)	*t*(40)	*p*
Happiness	3.22 (.89)	3.50 (.60)	1.20	.237
Sadness	2.34 (.52)	2.14 (.65)	.539	.593
Disgust	2.02 (.58)	1.99 (.72)	.154	.878
Anger	1.88 (.71)	1.80 (.66)	.345	.732
Fear	1.85 (.68)	1.69 (.41)	.888	.381
*Note*. ASD = autism spectrum disorder.				

**Table 2 tbl2:** Correlations Between Moral Acceptability Ratings for the Different Emotion categories

	Emotion	Happiness	Sadness	Fear	Disgust
Full sample	Sadness	−.085			
	Fear	−.659***	.527***		
	Disgust	−.228	.780***	.603***	
	Anger	−.528***	.766***	.797***	.802***
Control	Sadness	−.299			
	Fear	−.816***	.615**		
	Disgust	−.438*	.705***	.665***	
	Anger	−.719***	.779***	.907***	.787***
ASD	Sadness	.172			
	Fear	−.306	.469*		
	Disgust	.026	.826***	.572**	
	Anger	−.264	.766***	.674***	.819***
*Note*. ASD = autism spectrum disorder.					
* *p* < .05. ** *p* < .01. *** *p* < .001.					

**Table 3 tbl3:** Regression Models Predicting Global Morality Score, (A) Including Age, Gender, Depression, and Anxiety in the First Step, Alexithymia in the Second Step, and ASD Symptom Severity in the Third, (B) Including Demographic Variables in the First Step, ASD Symptom Severity in the Second Step, and Alexithymia in the Third

Step	Predictor	Control Global Morality score				ASD Global Morality score			
		β	*p*	*R*^2^	Δ*R*^2^ (*p*)	β	*p*	*R*^2^	Δ*R*^2^ (*p*)
A									
1	Age	−.281	.190	30.0%	30.0% (.172)	.086	.658	43.3%	43.3% (.018)
	Gender	−.242	.305			.155	.558		
	Depression	.333	.327			−.325	.299		
	Anxiety	.145	.687			−.421	.081		
2	Age	−.220	.157	65.8%	35.9% (.001)	.092	.649	43.4%	0.1% (.841)
	Gender	−.095	.579			.137	.634		
	Depression	.097	.695			−.321	.319		
	Anxiety	−.419	.168			−.424	.088		
	Alexithymia	.943	.001			.039	.841		
3	Age	−.235	.154	66.2%	0.4% (.678)	.116	.558	48.9%	5.4% (.183)
	Gender	−.107	.551			.093	.740		
	Depression	.075	.775			−.296	.347		
	Anxiety	−.407	.195			−.448	.068		
	Alexithymia	.875	.008			−.047	.815		
	ASD severity	.101	.678			.257	.183		
B									
									
1	Age	−.281	.190	30.0%	30.0% (.172)	.086	.658	43.3%	43.3% (.018)
	Gender	−.242	.305			.155	.558		
	Depression	.333	.327			−.325	.299		
	Anxiety	.145	.687			−.421	.081		
2	Age	−.332	.098	45.3%	15.3% (.050)	.121	.529	48.7%	5.4% (.173)
	Gender	−.245	.256			.076	.775		
	Depression	.129	.687			−.292	.340		
	Anxiety	−.001	.997			−.450	.059		
	ASD severity	.514	.050			.244	.173		
3	Age	−.235	.154	66.2%	20.9% (.008)	.116	.558	48.9%	0.2% (.815)
	Gender	−.107	.551			.093	.740		
	Depression	.075	.775			−.296	.347		
	Anxiety	−.407	.195			−.448	.068		
	ASD severity	.101	.678			.257	.183		
	Alexithymia	.875	.008			−.047	.815		
*Note*. ASD = autism spectrum disorder.
